# The unexpected survival of an ancient lineage of anseriform birds into the Neogene of Australia: the youngest record of Presbyornithidae

**DOI:** 10.1098/rsos.150635

**Published:** 2016-02-24

**Authors:** Vanesa L. De Pietri, R. Paul Scofield, Nikita Zelenkov, Walter E. Boles, Trevor H. Worthy

**Affiliations:** 1 Canterbury Museum, Rolleston Avenue, Christchurch 8013, New Zealand; 2 Borissiak Paleontological Institute, Russian Academy of Sciences, Moscow, Russia; 3 Australian Museum, Ornithology Section, Sydney, New South Wales, Australia; 4 School of Biological Sciences, Flinders University, Adelaide, South Australia 5001, Australia

**Keywords:** Miocene, fossil birds, *Wilaru tedfordi*, Gondwana, palaeobiogeography

## Abstract

Presbyornithids were the dominant birds in Palaeogene lacustrine assemblages, especially in the Northern Hemisphere, but are thought to have disappeared worldwide by the mid-Eocene. Now classified within Anseriformes (screamers, ducks, swans and geese), their relationships have long been obscured by their strange wader-like skeletal morphology. Reassessment of the late Oligocene South Australian material attributed to *Wilaru tedfordi*, long considered to be of a stone-curlew (Burhinidae, Charadriiformes), reveals that this taxon represents the first record of a presbyornithid in Australia. We also describe the larger *Wilaru prideauxi* sp. nov. from the early Miocene of South Australia, showing that presbyornithids survived in Australia at least until *ca* 22 Ma. Unlike on other continents, where presbyornithids were replaced by aquatic crown-group anatids (ducks, swans and geese), species of *Wilaru* lived alongside these waterfowl in Australia. The morphology of the tarsometatarsus of these species indicates that, contrary to other presbyornithids, they were predominantly terrestrial birds, which probably contributed to their long-term survival in Australia. The morphological similarity between species of *Wilaru* and the Eocene South American presbyornithid *Telmabates antiquus* supports our hypothesis of a Gondwanan radiation during the evolutionary history of the Presbyornithidae. *Teviornis gobiensis* from the Late Cretaceous of Mongolia is here also reassessed and confirmed as a presbyornithid. These findings underscore the temporal continuance of Australia’s vertebrates and provide a new context in which the phylogeny and evolutionary history of presbyornithids can be examined.

## 1. Introduction

The survival of Australia’s iconic vertebrate fauna during much of the Cenozoic is tightly linked to the continent’s extended period of geographical isolation [1]. Lineages that were once widely distributed across the globe but have survived, or did survive until recently, as Australian endemic relicts include marsupials, monotremes, ceratodontid lungfish, meiolaniid turtles and madtsoiid snakes [1–4]. Within Aves, the flamingo-like palaelodids that were widespread worldwide during the Neogene prevailed in Australia until *ca* 1 Ma [5], and the monospecific endemic family-level taxa Pedionomidae (plains-wanderer) and Anseranatidae (magpie goose; also present in New Guinea) are relicts of lineages that extended outside of Australia in the past [6–8]. Avian fossils from mid-Cenozoic localities, such as those from the Etadunna Formation in South Australia and Riversleigh in northwestern Queensland, have additionally underscored the remarkable temporal continuance of Australia’s endemic birds (e.g. [7,9–11]).

Representatives of the extinct anseriform (screamers, swans, ducks and geese) family-level taxon Presbyornithidae are mostly known from the Palaeocene and early Eocene of the Americas, particularly North America [12–18] (see also [19]). More recently, they were also described from the Palaeocene and early Eocene of Mongolia [20], from where they were previously known [21,22], and were also reported from the early and early middle Eocene of the Canadian High Arctic [23]. The presence of late Eocene–early Oligocene presbyornithids in Europe [24,25] has been disputed, as these remains are considered to represent those of phoenicopteriform birds (flamingos and relatives) [19,26,27]. A Late Cretaceous (*ca* 72–66 Ma) presbyornithid, *Teviornis gobiensis*, was described based on a complete carpometacarpus and some other isolated wing elements [28], but its presbyornithid, and even anseriform, affinities have been challenged [29] (see §4.1).

Evidence of presbyornithids in Australia has not yet been established, although fossils of ‘early-diverging’ anseriforms were reported from early Eocene deposits of the Tingamarra Local Fauna [30]. Elzanowski & Boles [30] noted that a coracoid from the same fauna previously referred to the ‘Graculavidae’ (form-taxon, see [31]) by Boles [32] may be a presbyornithid. An abundance of late Oligocene and some early Miocene remains of a putative stone-curlew (Charadriiformes: Burhinidae) from northern South Australia was first reported by Tedford *et al.* [33] and followed by Rich [34] and Vickers-Rich [35]. The material was then formally described by Boles *et al.* [36] and attributed to a new genus and species, *Wilaru tedfordi*. In this contribution, we show that *W. tedfordi* was a representative of the Presbyornithidae. Additionally, we describe an early Miocene species of *Wilaru*, showing that presbyornithids survived in Australia *ca* 25 Ma after the youngest fossil record for them elsewhere. We also provide the first example of successive taxa in an avian lineage in the Australian Oligo-Miocene. The palaeobiological, phylogenetic and evolutionary implications of these findings are further discussed.

## 2. Material and methods

Anatomical terminology follows Baumel & Witmer [37]. Measurements are in millimetres and were rounded to the nearest 0.1. Institutional abbreviations: CM, Canterbury Museum, Christchurch, New Zealand; MV, Museum Victoria, Melbourne, Australia; PIN, The Borissiak Paleontological Institute of the Russian Academy of Sciences, Moscow, Russia; SAM, South Australian Museum, Adelaide, South Australia, Australia; USNM, National Museum of Natural History, Washington DC, USA.

### 2.1 Fossil comparative material

The following specimens of *Presbyornis pervetus* were examined at USNM: skull—USNM 299846, USNM 618166, USNM 618202; premaxilla—USNM 510082, USNM 299845 (six nose slab); mandible—USNM 299847, USNM 618169, USNM 618215; quadrate—USNM 498770; thoracic vertebrae—USNM 616555, USNM 616556, USNM 618205, USNM 618207; sternum—USNM 618212, USNM 618214; scapula—USNM 616557–60; coracoid—USNM 618183, USNM 616561–67 (four sternal and three omal parts); humerus—USNM 483163 (cast), USNM 616568, USNM 618204, USNM 618180; ulna—USNM 616569–74; carpometacarpus—USNM 618168, USNM 618226, USNM 618227; femur—USNM 618228–35; tibiotarsus—USNM 483165 (cast), USNM 618192–96, USNM 618236; tarsometatarsus—USNM 483166 (cast), USNM 618175–76, USNM 618177, USNM 618178, USNM 618213, USNM 618237; pelvis—USNM 618167, USNM 618172, USNM 618198. Casts of *Presbyornis isoni* were examined at USNM and PIN. The material attributed to *T. gobiensis*, as well as the holotype of *Presbyornis mongoliensis* and the collection of *Presbyornis* sp. specimens from the early Eocene of Mongolia [20] were examined at PIN. *Telmabates antiquus* was assessed based on casts at PIN, as well as the original description and images [13] and on Ericson’s diagnosis of the species [17]. Lithornithidae (Palaeognathae) were also considered for comparative purposes.

### 2.2 Extant comparative material

The following specimens were examined: Anhimidae: *Anhima cornuta* (MV B.12574; USNM 345208); *Chauna chavaria* (PIN Osteology collection 43-2-1); *Chauna torquata* (CM Av.21208). Anseranatidae: *Anseranas semipalmata* (SAM B.36790; USNM 621019). Anatidae: *Anser caerulescens* (SAM B36868); *Biziura lobata* (CM Av.7116); *Cereopsis novaehollandiae* (CM Av.21198; SAM B39638, 49165); *Hymenolaimus malacorhynchos* (CM Av.5217); *Tadorna tadornoides* (SAM B.39583; 39872); *Tadorna variegata* (CM Av.12424). Burhinidae: *Burhinus grallarius* (SAM B.48793; B.49554); *Burhinus capensis* (MV B.13648); *Esacus magnirostris* (SAM B.5052).

Fossil and living phoenicopteriforms (flamingos and allies) were also examined.

### 2.3 Remarks on the material previously attributed to *Wilaru tedfordi* by Boles *et al*.

Paratype humeri AMNH 11407 and AMNH 11406 of the type species *W. tedfordi* are part of the same bone and now joined. Coracoid AMNH 11414 is a left one, contra Boles *et al.* [36]. Right coracoid SAM P.23625 is now a paratype of a new species (see below) and is here removed from the referred material of *W. tedfordi*. Scapulae SAM P.48923 (fig. in [36]), AMNH 10990, AMNH 11434 and AMNH 11477 are here removed from the referred material of *W. tedfordi*, as they are attributable to the Palaelodidae (Phoenicopteriformes). The unnumbered piece of mid-shaft associated with ulna AMNH 11456 is of a carpometacarpus, not an ulna. Distal ulna AMNH 10995 (not listed in [36]) may be associated with the unnumbered proximal right ulna mentioned by Boles *et al.* as they were found in the same box. Carpometacarpus AMNH 11474 (distal left) and AMNH 10998 (proximal left) are part of the same bone. Right carpometacarpus missing proximal end AMNH 10999 and proximal left carpometacarpus SAM P.42004 are here added to the referred material of *W. tedfordi*. AMNH 10986 was erroneously listed as a proximal tibiotarsus, it is a distal end and belongs to Psittaciformes; the proximal tibiotarsus considered by Boles *et al.* is AMNH 11416 (we note that this bone was not formally referred to *W. tedfordi*, and we consider it to be Aves indet.). UCMP57152, a distal left tibiotarsus, is removed from the referred material as it is referrable to *Australotadorna alecwilsoni*. Two specimens, a distal right tibiotarsus (SAM P.53134), from SAM North Locality, Lake Palankarinna, and a distal left tibiotarsus (SAM P.53135) from White Sands Basin, Lake Palankarinna, are here added to the referred material. The accompanying fragment of the left tarsometatarsus SAM P.48931 is a right proximal tarsometatarsus, not a left one.

## 3. Systematic palaeontology

### 3.1 Order Anseriformes Wagler, 1831

#### 3.1.1 Family Presbyornithidae Wetmore, 1926

In his revision of the New World fossil record of the Presbyornithidae, Ericson [17] recognized four species, *Telmabates antiquus* Howard, 1955 [13] from the early Eocene of southern South America (Chubut, Argentina), and three from the late Palaeocene to early Eocene of North America: *Presbyornis pervetus*Wetmore, 1926 [12], *Presbyornis isoni* Olson, 1994 [14] and *Presbyornis recurvirostra*(Hardy, 1959 [38]). Two species were later described from Mongolia: the Late Cretaceous *Teviornis gobiensis* Kurochkin, Dyke, & Karhu [28], and the early Eocene *Presbyornis mongoliensis* Kurochkin & Dyke [20].

#### 3.1.2 Attribution of *Wilaru tedfordi* to the Presbyornithidae

**Figure 1.**
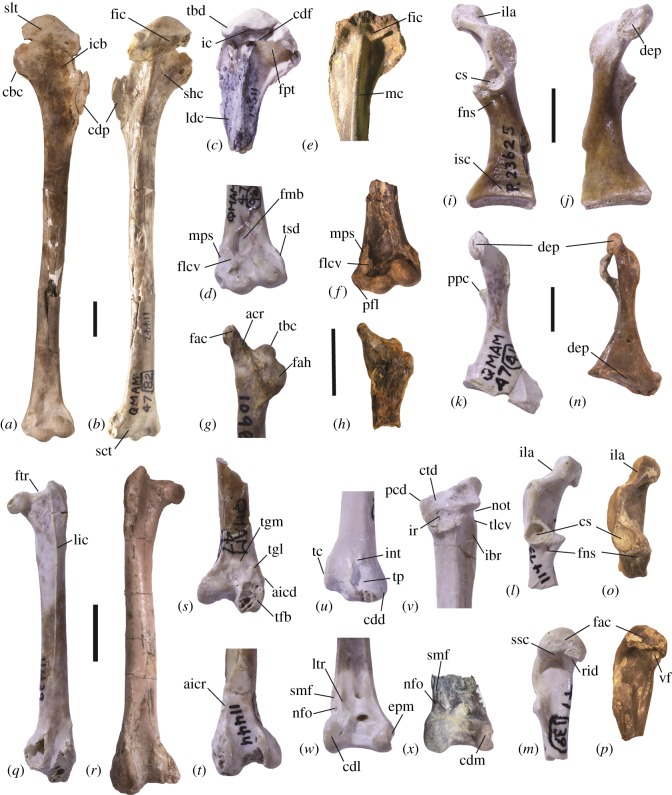
Postcranial elements of *Wilaru tedfordi* (*a*–*d*,*g*,*k*–*m*,*q*,*s*–*w*) and *W. prideauxi* sp. nov. (*i*,*j*) from the late Oligocene and early Miocene of Australia in comparison to *Presbyornis pervetus* (*e,f,h,n–p,r,x*) from the early Eocene of North America. (*a*,*b*) Left humerus of *W. tedfordi* (holotype SAM P.48925) in cranial and caudal views; (*c*) proximal left humerus (paratype AMNH 1151) of *W. tedfordi* in caudal view; (*d*) distal left humerus (paratype AMNH 11452) of *W. tedfordi*in caudal view. (*e*) Proximal left and distal left, (*f*) humerus of *P. pervetus* in caudal (USNM 618204) and cranial (USNM 618180) views, respectively. (*g*) Right scapula (AMNH 10989) of *W. tedfordi* in lateral view; (*h*) left scapula of *P. pervetus* (USNM 618223) in medial view. (*i*,*j*) Right coracoid of *W. prideauxi* sp. nov (paratype SAM P.23625) in dorsal and ventral views. (*k*) Left coracoid (AMNH 11426) of *W. tedfordi* in ventral view; (*l*,*m*) left coracoid, omal extremity (AMNH 11473) of *W. tedfordi* in dorsal and medial views. (*n*,*o*,*p*) Left coracoid of *P. pervetus* in ventral (USNM 618183), dorsomedial (USNM 616565) and medial (USNM 616565) views. (*q*) Left femur (AMNH 11439) of *W. tedfordi* in cranial view; (*r*) right femur of *P. pervetus* (USNM 618228) in cranial view. (*s*,*t*) Left distal femur (AMNH 11444) of *W. tedfordi* in caudal and cranial views. (*u*) Left distal (AMNH 10995) and (*v*) right proximal (AMNH 11457) ulna of *W. tedfordi* in caudal and ventral views. (*w*) Distal right tibiotarsus (AMNH 11440) of *W. tedfordi* in cranial view. (*x*) Distal right tibiotarsus of *P. pervetus* (USNM 618236) in cranial view. *Abbreviations:* acr, acromion; aicd, impressio ansae m. iliofibularis, pars caudalis; aicr, impressio ansae m. iliofibularis, pars cranialis; cbc, crista bicipitalis; cdf, crus dorsale fossae; cdl, condylus lateralis; cdm, condylus medialis; cdp, crista deltopectoralis; cs, cotyla scapularis; ctd, cotyla dorsalis; dep, depression; epm, epicondylus medialis; fac, facies articularis clavicularis; fah, facies articularis humeralis; fic, fossa at incisura capitis; flcv, facet for lig. collat. ventrale; fmb, fossa m. brachialis; fns, foramen nervi supracoracoidei; fpt, fossa pneumotricipitalis; ftr, fossa trochanteris; ibr, impressio brachialis; ic, incisura capitis; icb, impressio coracobrachialis; ila, impressio lig. acrocoracohumeralis; int, incisura tendinosa; ir, incisura radialis; isc, impressio m. sternocoracoidei; ldc, scar for m. latissimus dorsi caudalis; lic, linea intermuscularis cranialis; ltr, lateral tuberositas retinaculi extensoris; mc, margo caudalis; mps, scar for m. pronator superficialis; nfo, nutrient foramen; not, notch; pcd, processus cotylaris dorsalis; pfl, processus flexorius; ppc, processus procoracoideus; rid, ridge; sct, sulcus scapulotricipitalis; shc, scar for m. scapulohumeralis cranialis; slt, sulcus lig. transversus; smf, sulcus m. fibularis; ssc, sulcus m. supracoracoidei; tbd, tuberculum dorsale; tc, tuberculum carpale; tfb, trochlea fibularis; tgl, tuberculum m. gastrocnemialis lateralis; tgm, tuberculum m. gastrocnemialis medialis; tlcv, tuberculum lig. collateralis ventrale; tsd, tuberculum supracondylare ventrale; tvc, tuberculum coracoideum; vf, ventral fossa. Scale bar is 10 mm. (*c*–*f*,*l*–*m*,*o*–*p*,*u*–*x*) not to scale.

**Figure 2.**
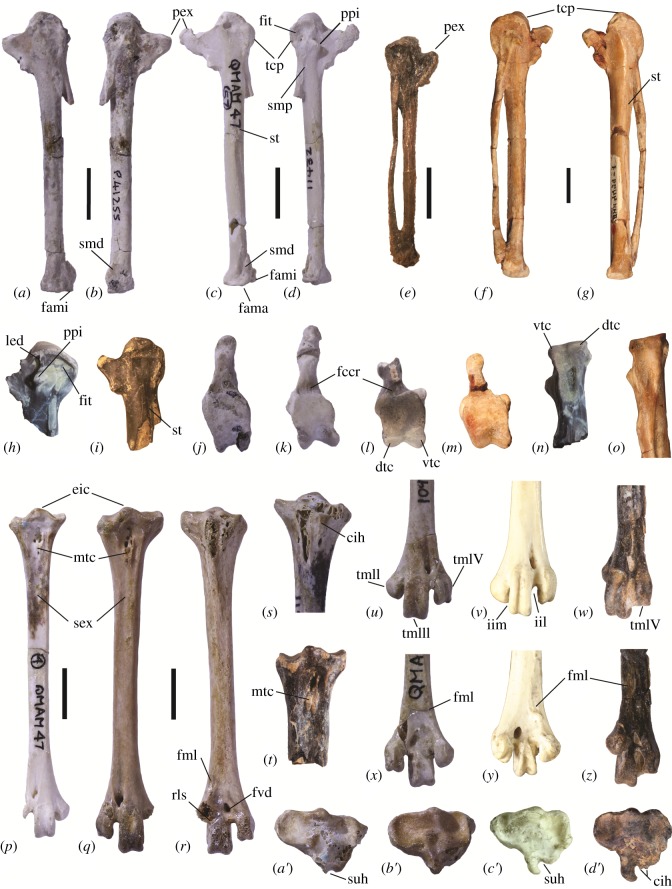
Postcranial elements of *Wilaru tedfordi* (*c*,*d*,*k*,*p*,*s*,*u*,*x*,*a*^′^) and *W. prideauxi* sp. nov. (*a,b,j,q,r,b^′^*) from the late Oligocene and early Miocene of Australia in comparison to *Presbyornis pervetus* (*e,h,i,l,n,t,w,z,d*^′^) from the early Eocene of North America, *T. gobiensis* from the Late Cretaceous of Mongolia (*f*,*g*,*m*,*o*), and the extant anhimid *Chauna torquata* (*v*,*y*,*c*^′^). (*a*,*b*,*j*) Left carpometacarpus (paratype SAM P.41255) of *W. prideauxi* in dorsal, ventral and proximal views. (*c*,*d*,*k*) Left carpometacarpus (AMNH 11432) of *W. tedfordi* in dorsal, ventral and proximal views. (*e*) Left carpometacarpus of *P. pervetus*(USNM 618168). (*f*,*g*,*m*,*o*) Right carpometacarpus (reversed; PIN 44991-1) of *T. gobiensis* in ventral, dorsal, proximal and caudal views. (*h*,*l*,*n*) Right carpometacarpus of *P. pervetus* (USNM 618227) in ventral, proximal and caudal views (l has been reversed). (*i*) Left proximal carpometacarpus of *P. pervetus* (USNM 618226) in dorsal view. (*p,s,a^′^*), right tarsometatarsus (AMNH 11413) of *W. tedfordi* in dorsal, proximal plantar and proximal views. (*q,r,b*^′^) Right tarsometatarsus (holotype SAM P.53136) of *W. prideauxi* in dorsal, plantar and proximal views. (*t,d*^′^) Proximal right tarsometatarsus of *P. pervetus* (USNM 618178) in dorsal and proximal views. (*u*,*x*) Distal left tarsometatarsus (AMNH 10980) in dorsal and plantar views. (*v,x,c*^′^) Right tarsometatarsus (CM Av.21208; V and Y reversed) of *C. torquata* in dorsal, plantar and proximal views. (*w*,*z*) Distal right (reversed) tarsometatarsus of *P. pervetus* (USNM 618213) in dorsal and plantar views. cih, crista(e) intermedia(e) hypotarsi; dtc, dorsal rim of trochlea carpalis; eic, eminentia intercotylaris; fami, facies articularis digitalis minor; fama, facies articularis digitalis major; fccr, fovea carpalis cranialis; fit, fossa infratrochlearis; fmI, fossa metatarsi I; fvd, foramen vasculare distale; iil, incisura intertrochlearis lateralis; iim, incisura intertrochlearis medialis; led, ledge; mtc, tuberositas m. tibialis cranialis; pex, processus extensorius; ppi, processus pisiformis; rls, rounded ligamental scar; sex, sulcus extensorius; smd, synostosis metacarpalis distalis; smp, synostosis metacarpalis proximalis; st, sulcus tendineus; tcp, trochlea carpalis; tmII, trochlea metatarsi II; tmIII, trochlea metatarsi III; tmIV, trochlea metatarsi IV; vtc, ventral rim of trochlea carpalis. Scale bar is 10 mm. (*h*–*o*, *s*–*d*′) Not to scale. Note that the carpometacarpi of *P. pervetus* vary greatly in size (from *Wilaru*-sized to as shown [17]).

*Wilaru tedfordi* Boles, Finch, Hofheins, Vickers-Rich, Walters, & Rich [36] is here assigned to the Presbyornithidae based on the following features of the holotype (a left humerus SAM P48925, formerly AMNH 11442; figure 1*a*,*b*) and paratypes (all humeri; figure 1*c*,*d*), which, combined, are not present in any extant charadriiform lineage, and are characteristic of presbyornithids (most characters are based on [13,17]): (1) humerus elongated in relation to the width of its proximal and distal ends, with a straight shaft in caudal and cranial views (figure 1*a*,*b*); (2) fossa pneumotricipitalis deep, non-pneumatic, and dorsoventrally very wide (figure 1*b*,*c*); (3) fossa pneumotricipitalis dorsalis absent (contra [36]); (4) crus dorsale fossae continuous (or nearly continuous) with margo caudalis (figure 1*b*,*c*; see §3.2.1); (5) crus dorsale fossae transverse and delimiting distally a deep fossa in the incisura capitis (figure 1*b*,*c*; this feature was not mentioned in [17]); (6) well-marked and elongated insertion scar for m. scapulohumeralis cranialis (figure 1*b*); (7) scar for m. latissimus dorsi caudalis elongated, ending distally at point of junction of crista deltopectoralis and corpus humeri (figure 1*c*); (8) tuberculum dorsale wider than long, markedly elevated above surface beside it (figure 1*c*); (9) impressio coracobrachialis shallow (but rounded and large; figure 1*a*); (10) sulcus lig. transversus restricted to ventral portion of humerus (i.e. not extending dorsally, figure 1*a*; character after [36]); (11) sulcus n. coracobrachialis absent; (12) crista deltopectoralis long, with more than half of its length distal of crista bicipitalis (figure 1*a*,*b*); (13) sulcus scapulotricipitalis indistinct (figure 1*b*); (14) impressio m. pronator superficialis and attachment surface for lig. collaterale ventrale adjacent (figure 1*d*, see amended diagnosis below); (15) tuberculum supracondylare dorsale poorly developed (figure 1*d*).

Boles *et al.* [36] assigned *W. tedfordi* to Burhinidae based on characters (2), (9) and (15). We note that the fossa pneumotricipitalis in burhinids is not as dorsoventrally wide as that of presbyornithids (character 2), and that the tuberculum supracondylare dorsale is better developed in burhinids, as noted in [36] (character 15). Among the characters here presented, Boles *et al.* also noted that *W. tedfordi* differs from burhinids in (1, shaft not straight in burhinids), (4), (5) and (10), and we further note that they differ in (6), (7), (8), (11) and (13). Contra Boles *et al.* [36], a crus dorsale fossae is present, and a fossa pneumotricipitalis dorsalis is absent.

Our attribution of the referred material (coracoids, scapulae, ulnae, carpometacarpi, femora, tibiotarsi and tarsometatarsi) of *W. tedfordi* to the Presbyornithidae is based on Ericson’s [17] diagnosis for the family and our observations (see §§i and i).

#### 3.1.3 Remarks on charadriiform affinities

The skeletal morphology of burhinids is possibly derived within Charadriiformes [39], and some of the features mentioned by Boles *et al.* [36] do occur in burhinids but not in most other charadriiforms. A similarity between the postcranial skeleton of presbyornithids and burhinids had been noted in the past (e.g. [40]). The ecological disparity within Charadriiformes has resulted in a wide array of morphological variation, although it is unlikely that burhinids represent the ancestral charadriiform morphology [41,42]. Presbyornithids have been linked, primarily based on postcranial morphology, to both flamingos (Phoenicopteriformes) and shorebirds (Charadriiformes) (e.g. [13,43]). The type species *P. pervetus* was originally considered to be a recurvirostrid [12] and it was not until the mid-1970s when additional cranial material was assessed that anseriform affinities were proposed [24,40,44] (see [19] for a review).

### 3.2 Genus *Wilaru* Boles, Finch, Hofheins, Vickers-rich, Walters, & Rich, 2013

*Type species: Wilaru tedfordi*Boles, Finch, Hofheins, Vickers-Rich, Walters, & Rich, 2013

*Type locality and age:*Lake Pinpa (=Pine Lake), Site C, South Australia; Namba Formation, late Oligocene (24–26 Ma).

#### 3.2.1 Amended diagnosis

We here focus on features that differentiate presbyornithid genus-level taxa. Most presbyornithid characters mentioned by Howard [13] and Ericson [17] are not repeated here but newly defined or identified features are. Apart from those mentioned above for Presbyornithidae, *Wilaru* is further characterized by the following combination of features:

Humerus with (1) dorsal crus continuous with margo caudalis (figure 1*c*; as in *Telmabates*); (2) incisura capitis undercuts caput humeri and tuberculum ventrale (figure 1*c*; as in *Telmabates*, only caput humeri in *Presbyornis*); (3) fossa m. brachialis diagonally oriented, elongated and ovoid (more elongate in *Wilaru* compared to *Presbyornis*; figure 1*d*,*f*); (4) processus flexorius protruding ventrally (figure 1*d*; less pronounced than in *Presbyornis*, figure 1*f*); (5) scars of m. pronator superficialis and lig. collaterale ventrale adjacent, and reaching to about same level proximally (figure 1*d*; as in *Telmabates*, impressio m. pronator superficialis reaches farther proximally in *Presbyornis*, figure 1*f*); (6) position of tuberculum supracondylare dorsale craniocaudally more distal in relation to tuberculum supracondylare ventrale (tuberculum supracondylare dorsale extends proximally of tuberculum supracondylare ventrale in *Presbyornis*); (7) size of m. pronator superficialis attachment only slightly smaller than that of the lig. collaterale ventrale (much smaller in *Presbyornis*; figure 1*d*,*f*).

Coracoid (8) overall stouter and more robust compared to other presbyornithids (varies within species of *Wilaru*, figure 1*i*–*k*; see below); (9) with processus procoracoideus short with rugose tip for ligamental attachment (figure 1*k*), and lacking pronounced mediocranial projection (processus procoracoideus long in *Presbyornis*, figure 1*n*); (10) with ventral fossa in sulcus m. supracoracoidei absent (figure 1*p*; present in *Presbyornis*, but shallow in *Telmabates*); (11) with transverse linear ridges within the impressio sternocoracoidei (figure 1*i*; as in all presbyornithids).

Scapula with (12) elongated and pointed acromion (figure 1*g*, as in *Presbyornis*, figure 1*h*, less so in *Telmabates*); (13) facies articularis clavicularis forms sharp crest projecting laterally from acromion (figure 1*g*); (14) facies articularis humeralis only slightly longer than deep (i.e. round) and slightly concave (figure 1*g*); (15) base of acromion markedly separated from tuberculum coracoideum (figure 1*g*, as in *Telmabates*, softer transition, i.e. broader base, in *Presbyornis*; figure 1*h*).

Ulna with (16) pronounced notch in cranial view, between cotyla ventralis and tuberculum ligamenti collateralis ventralis (figure 1*v*; not mentioned in [38], as in presbyornithids); (17) tuberculum ligamenti collateralis ventralis elongated and convex ventrally (figure 1*v*; as in all anseriforms); (18) impressio brachialis very deep (figure 1*v*); (19) cotyla dorsalis with processus cotylaris dorsalis short and draped on cranial facies with dorsodistal margin continuing as a ridge to ligamental insertions that distally close the incisura radialis (figure 1*v*; more elongate and with ligament insertion scars in incisura radialis less marked in *Presbyornis*); (20) tuberculum carpale (figure 1*u*) well developed with flattened ventral margin (as in *Presbyornis*) but elongated in proximodistal direction (as in *Telmabates*); (21) depressio radialis poorly marked (as in *Telmabates*); (22) well-marked tendinal pit but incisura tendinosa very short, marked by short ridge dorsally (figure 1*u*; as in presbyornithids); (23) contour of condylus dorsalis in ventral view meets shaft at gradual angle (figure 1*u*; as in *Telmabates*, whereas it does so abruptly in *Presbyornis*).

Carpometacarpus with (24) trochlea carpalis relatively short ending level with distal side of processus pisiformis, as seen in ventral view (figure 2*d*); (25) area immediately cranial of processus pisiformis shallowly excavated (figure 2*d*; as in *Telmabates*, deeper in *Presbyornis*, figure 2*h*); (26) both rims of the trochlea carpalis extend caudally (figure 2*j*–*m*) and distally (figure 2*n*,*o*) to about same level (as in *Telmabates*); (27) sulcus tendineus (dorsal aspect) extends nearly to the synostosis metacarpalis proximalis, just about to distal end of the insertions scar of m. extensor metacarpi ulnaris (flexor attachment) (figure 2*c*,*i*; as in all presbyornithids); (28) processus extensorius with proximal margin straight, at right angles to long axis, and elongate cranially (figure 2*a*–*d*; as in *Telmabates*, shorter and more proximally oriented in *Presbyornis*, figure 2*e*,*i*); (29) synostosis metacarpalis proximalis longer than it is craniocaudally wide (figure 2*d*); (30) synostosis metacarpalis distalis relatively short (figure 2*d*, as in *Telmabates*; longer in *Presbyornis*, figure 2*e*); (31) facies articularis digitalis minor considerably smaller than facies articularis digitalis major in distal view (as in *Telmabates*; contrary to *Presbyornis* [17]).

Femur with (32) fossa trochanteris present (figure 1*q*); (33) pretrochanteric surface deeply concave (as in presbyornithids); (34) linea intermuscularis cranialis very prominent and separated from lateral margin of crista trochanteris (figure 1*q*; hardly distinguishable from crista trochanteris in *Presbyornis*, figure 1*r*); (35) round and papilla-like tuberculum m. gastrocnemialis lateralis with pars medialis situated medial to it on edge of fossa poplitea (figure 1*s*; as in *Telmabates*; tuberculum proximodistally elongate and less elevated in *Presbyornis*); (36) impressio ansae m. iliofibularis caudalis on lateral facies (figure 1*s*), distal to tuberculum m. gastrocnemialis lateralis, large and well marked with pit at distal margin (less marked in *Presbyornis*); (37) ansae m. iliofibularis cranialis prominent on craniolateral margin (figure 1*t*); (38) trochlea fibularis in caudal aspect relatively wide (figure 1*s*; as in *Telmabates*; smaller in *Presbyornis*).

Tibiotarsus with (39) epicondylus medialis well developed and medially prominent (figure 1*w*; as in *Telmabates*; less developed in *Presbyornis*); (40) lateral tuberositas retinaculi extensoris long (figure 1*w*); (41) well-marked depressio epicondylaris medialis (as in *Telmabates*; less so in *Presbyornis*); (42) well-marked sulcus m. fibularis opening laterocranially (unlike in all other anseriforms including *P. pervetus*, in which the sulcus faces entirely cranially) and continuing to the most distal part of the tuberositas retinaculi extensoris lateralis (figure 1*w*; as in *Telmabates*; sulcus ends more proximal in *Presbyornis*, figure 1*x*); (43) a nutrient foramen located distal to the sulcus m. fibularis and proximolateral of condylus lateralis (figure 1*w*,*x*; as in presbyornithids); (44) condylus medialis in cranial view much smaller than condylus lateralis (figure 1*w*,*x*; as in all presbyornithids).

Tarsometatarsus with (45) insertion areas for m. tibialis cranialis fused, with a distal (neuro-) vascular opening (figure 2*q*; two adjacent insertions and no distal opening in *Presbyornis*, figure 2*t*); (46) trochleae metatarsorum mediolaterally widely splayed, with trochlea metatarsi IV and II diverging markedly laterally and medially, respectively, from the shaft (figure 2*u*; trochleae little splayed in *Presbyornis*, figure 2*w*); (47) trochlea metatarsi II slightly shorter than trochlea metatarsi IV (figure 2*u*; in *P. pervetus* markedly retracted proximally and ending level with the intertrochlear notch, figure 2*z*); (48) fossa metatarsi I short and shallow (figure 2*r*,*x*; better marked in *Presbyornis* and *Telmabates*, figure 2*z*); (49) lateral margin of trochlea metatarsi IV diverging from shaft and then abruptly turning distally, displaying a sharp angle (figure 2*u*, as in *Telmabates* and *Presbyornis*, figure 2*w*); (50) trochlea metatarsi II lacking groove (figure 2*w*, as in presbyornithids). Note: tarsometatarsi of *T. antiquus* are only poorly preserved and not much detail can be inferred [13,17].

### 3.3 *Wilaru prideauxi* sp. nov.

*Holotype:* Right tarsometatarsus SAM P.53136 (formerly UCMP 108052) (figure 2*q,r,b*^′^).

*Type locality and age:* Leaf Locality, Lake Ngapakaldi, South Australia (UCMP locality V6213); Wipajiri Formation; Kutjamarpu Local Fauna; *ca* 23.4–22 Ma [45,46].

*Etymology:* After vertebrate palaeontologist Gavin Prideaux (1969–), who has worked extensively on Oligo-Miocene mammalian faunas from South Australia, including the formations bearing fossils of species of *Wilaru*.

*Paratypes:* Right coracoid SAM P.23625 (figure 1*i*,*j*), Mammalon Hill, Lake Palankarinna, South Australia; Etadunna Formation Zone D; Ngama Local Fauna; *ca* 22 Ma [45]. Left carpometacarpus SAM P.41255 (figure 2*a*,*b*), Mammalon Hill, Lake Palankarinna, South Australia.

*Measurements* (*mm*): Coracoid: medial length: 38.9; length from angulus medialis to proc. procoracoideus: 24.7; length from cotyla scapularis to processus acrocoracoideus: 19.6; maximum omal length of sulcus m. supracoracoideus: 11.1 (shorter in Phoenicopteriformes); depth of facies articularis clavicularis: 8.8; length of facies articularis humeralis: 12.8. Carpometacarpus (table 1). Tarsometatarsus: maximum length: 65.9; proximal width: 12.7; shaft width (mid-shaft): 4.7; shaft depth: 4.7; width of trochlea metatarsi III: 4.9; depth of trochlea metatarsi III: 6.9.

**Table 1.** Measurements of the carpometacarpi of *W. tedfordi* and *W. prideauxi* sp. nov.^†^. The distal width is measured across the articular surfaces (facies articularis digitalis major and facies articularis digitalis minor). Some specimens (italics), which tend to be only slightly larger than the rest, display a conspicuous, rugose, cranial enlargement on the processus extensorius (estimated in proximal width) (figure 2*c*,*d*,*k*). The presence of a well-developed carpal knob in some but not other specimens may indicate both age and sex differences. Those with a much elongated extensor process are likely to be males (§4.2).

			width of		
	maximum	proximal	proximal	distal	distal
	length	width	synostosis	width	depth
*AMNH 11432*	*54.2*	*15.2*	*5.7*	*6.8*	*5.1*
AMNH 11401	—	12.7	5.6	—	—
AMNH 10998	49.7	13.1	5.5	5.4	5.1
AMNH 11462	—	13.70	5.7	—	—
*AMNH 11448*	*54.8*	*15.3*	*5.7*	—	—
SAM P.48928	50.9	13.1	5.5	6.8	5.5
AMNH 11460	52.1	12.6	5.4	7.1	—
AMNH 11467	53.8	13.7	—	7.0	5.6
AMNH 10962	—	13.7	5.6	—	5.5
*SAM P.42004*	—	*14.3*^*a*^	*6.2*	—	—
SAM P.41255^†^	56.3	13.9	6.4	7.4	5.9

^a^Specimen SAM P.42004 is overall worn and has a slightly broken trochlea carpalis so its proximal width is likely to have been greater. The processus extensorius is nevertheless well developed.

*Differential diagnosis:*Only slightly larger than *W. tedfordi* but considerably stouter (figure 1*i*–*j*; figure 2*a*,*b*,*q*,*r*). Differs from *W. tedfordi* in: tarsometatarsus with (i) sulcus extensorius shallower (figure 2*q*); (ii) plantarly, rounded ligamental scar between trochleae metatarsi II and IV deeper and closer to foramen vasculare distale (figure 2*r*); (iii) fossa metatarsi nearly absent (figure 2*r*). Carpometacarpus with (iv) synostosis metacarpalis distalis proximodistally shorter (figure 2*a*,*b*); (v) facies articularis digitalis minor projecting further distally (figure 2*b*).

#### 3.3.1 Description and comparisons

*Coracoid*. As in all presbyornithids, a small foramen nervi supracoracoidei nearly adjacent to the cotyla scapularis is present (figure 1*i*,*l*,*o*), the impressio lig. acrocoracohumeralis is distinctly excavated on its medial side (figure 1*i*,*l*,*o*), there is an elongated depression for a ligamental attachment on the ventral side of the brachial tuberosity (figure 1*j*,*l*,*o*), and the cotyla scapularis is round and very deep (figure 1*i*,*l*,*o*). In *P. pervetus*, the facies articularis clavicularis markedly overhangs the shaft ventrally and encloses therein a distinct fossa ventral to the sulcus m. supracoracoidei (figure 1*p*). This fossa is absent in species of *Wilaru* (figure 1*m*). It is also absent in some of the Eocene presbyornithid specimens from Mongolia [20], and may only be very shallow in *T. antiquus*[13]. As in presbyornithids, the sulcus m. supracoracoidei is excavated under the dorsal part of the facies articularis clavicularis (figure 1*o*,*p*). The ventral shaft margin of the sulcus is thickened and rounded, ventrally a ridge is also present but contrary to species of *Presbyornis*, it overlaps the ventral profile in medial view (figure 1*m*). Transverse linear ridges within the impressio sternocoracoidei are present (figure 1*i*), as in *W. tedfordi* and all Anseriformes. As in *W. tedfordi* and *T. antiquus*, the ventral surface of the sternal end lacks the depression that is observed in species of *Presbyornis*(figure 1*n*).

The coracoid of *W. prideauxi* is stouter than that of *W. tedfordi*(cf. figure 1*i*,*j* with figure 1*k*), which in turn is only slightly stouter than that of *P. pervetus* (figure 1*n*). Presbyornithids are characterized by having the neck of the shaft narrow [17], whereas it is much broader in the superficially similar phoenicopteriforms (flamingos and palaelodids). Similar to species of *Presbyornis*, *Palaelodus ambiguus* bears a distinct fossa ventral to the sulcus m. supracoracoidei, which, as noted, is absent in species of *Wilaru*. Palaelodids are further distinguished from species of *Wilaru* by having a cranially directed processus procoracoideus (right angles to axis in *Wilaru*), a foramen n. supracoracoidei that is closer to the margin of the cotyla scapularis, and a much smaller impressio sternocoracoidei restricted to the sternal third of the length from the cotyla scapularis.

*Carpometacarpus*. The bone (figure 2*a*,*b*) is overall more robust than that of *W. tedfordi* (figure 2*c*,*d*), but many features are worn. The area immediately cranial of the processus pisiformis is shallowly excavated and the fossa infratrochlearis is shallow and limited to the area proximal of the processus pisiformis (figure 2*c*). In *P. pervetus* there is a proximally directed ledge separating the processus extensorius from the fossa infratrochlearis, which extends caudally past the processus pisiformis (figure 2*h*). The fovea carpalis cranialis is deeper in *P. pervetus* (figure 2*l*) than in species of *Wilaru* (figure 2*j*,*k*). As in *W. tedfordi* and *P. pervetus*, both facies of the trochlea carpalis are smaller than in the Cretaceous *T. gobiensis*, in which they extend more cranially (figure 2*f*,*g*). As in all presbyornithids, caudally the dorsal and ventral rims of the trochlea carpalis have equal distal extension (figure 2*j*–*m*), and the sulcus tendineus is elongate, proximally nearly reaching the scar for the insertion of m. extensor carpi ulnaris (figure 2*c*,*i*). The sulcus tendineus is also particularly elongated in *T. gobiensis* and screamers (Anhimidae), whereas it is somewhat intermediate in length between these taxa and anatids (ducks, geese and swans) in the magpie goose *Anseranas semipalmata*.

The synostosis metacarpalis distalis (figure 2*a*,*b*) is relatively shorter than that of *W. tedfordi*, being considerably longer in *P. pervetus*(figure 2*e*). The facies articularis digitalis minor has equal distal extent to the facies articularis digitalis major and so it projects further distally (figure 2*b*) than in *W. tedfordi*(figure 2*d*). In *W. tedfordi* and *T. antiquus*, the facies articularis digitalis minor ends slightly proximally of the facies articularis digitalis major (figure 2*c*,*d*). Further detail of the distal end is obscured by wear but does not seem to differ from that of *W. tedfordi*.

*Tarsometatarsus*. The proportions of the tarsometatarsus of species of *Wilaru*[36] differ greatly from those of *P. pervetus* in being much shorter and stouter [12]. The length of the tarsometatarsus is, however, not known for other species of presbyornithids. Within anseriforms, the tarsometatarsus of species of *Wilaru* most closely resembles that of anhimids, which they resemble in overall relative proportions (figure 2*q*,*r*), morphology of the hypotarsus (figure 2*a*^′^,*b*^′^,*d*^′^) and configuration of the distal trochleae (figure 2*u*–*y*).

At least one sulcus hypotarsi is present in species of *Wilaru*; the crista medialis hypotarsi is missing from specimens of both *W. tedfordi* and *W. prideauxi*(figure 2*a*^′^,*b*^′^). The hypotarsus is overall reduced compared to that of *P. pervetus*(figure 2*c*^′^), which has four hypotarsal ridges (as does *T. antiquus* [13]), with the crista medialis hypotarsi being well marked and the other three cristae less so. In species of *Wilaru*, other than the missing crista medialis hypotarsi, there is one well-marked crista intermedia hypotarsi (figure 2*s*) and the rest have been reduced to a flat embossment in the lateral portion of the hypotarsus. A similar condition can be observed the anhimid *C. torquata*, although in *A. cornuta* the large crista medialis hypotarsi is separated from a much smaller crista lateralis hypotarsi by two very low cristae intermediae hypotarsi which altogether form a triangular hypotarsus. As in *P. pervetus*, the cristae lateral to the medial crest are of similar small size and reach equally distally (figure 2*s*). The eminentia intercotylaris is not especially prominent proximally in species of *Wilaru*, but it is more prominent dorsally (figure 2*p*,*q*,*a*^′^). The mid-shaft depth equals its width in *W. prideauxi*, whereas in *W. tedfordi* its width slightly exceeds its depth.

At the distal end, the trochlea metatarsi II is lacking so it is not known whether a medial groove was present. Within Anseriformes, this groove is absent in presbyornithids (including *W. tedfordi*, figure 2*u*), anhimids (figure 2*v*), and anseranatids (magpie goose). In *P. pervetus* (figure 2*w*,*z*) and *P. mongoliensis*, the trochleae are more narrowly splayed than they are in species of *Wilaru*, where their divergence is similar to that of anhimids (figure 2*v*) or *A. semipalmata*. The incisura intertrochlearis medialis and the incisura intertrochlearis lateralis extend equally proximally in species of *Wilaru*, whereas in both anhimids and *A. semipalmata* the medial notch is shallower proximally (figure 2*v*). Therefore, trochlea metatarsi II has less distal extent than trochlea metatarsi IV (figure 2*u*). In *W. tedfordi*, the trochlea metatarsi II is only slightly retracted plantarly so in distal view, most of its depth overlaps the trochlea metatarsi III, and thus is less retracted than in anhimids and *A. semipalmata* but similar to the terrestrial Cape Barren goose *C. novaehollandiae*. The fossa metatarsi I is barely perceptible in *W. prideauxi*(figure 2*r*), but it is better marked in *W. tedfordi*(figure 2*x*), in which it is situated much lower compared with *P. pervetus*(figure 2*z*).

## 4. Results and discussion

Crown-group Anseriformes comprise three extant family-level taxa: the South American screamers (Anhimidae), the magpie goose (Anseranatidae) of Australia and New Guinea, and the cosmopolitan Anatidae (ducks, swans and geese). A sister group relationship between Anhimidae and the clade (Anseranatidae + Anatidae) is supported by molecular and morphological evidence (e.g. [47,48]). Presbyornithids have been recovered as the sister taxon to Anatidae in cladistic analyses [40,47], but the character evidence supporting this relationship is weak [19]. These studies have been primarily based on the morphology of *P. pervetus*, and the possibility that some features of this taxon are derived within Presbyornithidae [19] has not been fully explored. Some of the similarities of species of *Wilaru* (and *T. antiquus*) to anhimids provide a new context in which the palaeobiology and evolutionary history of presbyornithids can be examined.

### 4.1 *Teviornis gobiensis*, a Cretaceous presbyornithid

*Teviornis gobiensis*, known primarily from its carpometacarpus (figure 2*f*,*g*,*m*,*o*), was attributed to the Anseriformes mainly based on its straight os metacarpale minus, and to the Presbyornithidae based on (i) the caudal part of the dorsal rim of the trochlea carpalis being well developed and connecting with the dorsal edge of the os metacarpale majus, (ii) the presence of well-developed scars for lig. ulnocarpometacarpale dorsale (fossa supratrochlearis) and lig. radiocarpometacarpale dorsale (fossa infratrochlearis), and (iii) the presence of a small canalis interosseus distalis in the fossa infratrochlearis [28]. Clarke & Norell [29] challenged presbyornithid, and even anseriform affinities of *T. gobiensis*, noting that of the diagnostic characters listed a straight minor metacarpal may be a plesiomorphic feature of Neornithes, that features (ii) and (iii) are present in other anseriforms, and (i) is also present in anhimids. Clarke and Norell did not, however, consider all the distinguishing features mentioned in the description. A recent study [49] supported anseriform affinities of this taxon, noting marked differences from other taxa with a non-curved carpometacarpus (e.g. Gallinuloididae, Lithornithidae). Additional features confirm the identity of *T. gobiensis* as a presbyornithid, namely (iv) the dorsal and ventral rims of the trochlea carpalis extend caudally and distally to about same level, (v) the sulcus tendineus is very elongate, extending just about to the distal end of the scar for M. extensor carpi ulnaris, (vi) in distal view, the facies articularis digitalis minor is considerably smaller than the facies articularis digitalis major (as in *Wilaru* and *Telmabates*), and (vii) the synostosis metacarpalis proximalis is longer than it is craniocaudally wide. Within anseriforms, feature (iv) is present only in presbyornithids, (v) in presbyornithids and anhimids, (vii) is present in presbyornithids, anhimids, anseranatids, and only few anatids, whereas (vi) is widely distributed within Anseriformes but the alternate condition is present in *P. pervetus*.

Kurochkin *et al.* [28] noted that *T. gobiensis* differed from other presbyornithids in having a fossa infratrochlearis stretched markedly craniocaudally. A shallow, craniocaudally elongated fossa is nonetheless also present in species of *Wilaru* (figure 2*d*). Similarly, the dorsoventrally and craniocaudally widened proximal portion of the os metacarpale minus are present in species of *Wilaru* but also in anhimids and other anseriforms, suggesting they could be plesiomorphic features for Presbyornithidae that are absent in *P. pervetus*. *T. gobiensis*, therefore, displays a combination of features of the carpometacarpus present uniquely in presbyornithid genus-level taxa.

### 4.2 Palaeobiology of species of *Wilaru*: terrestrial and territorial

The cranially elongated extensor process of the carpometacarpus of some specimens of *W. tedfordi* forms a conspicuous rugose enlargement (figure 2*c*,*d*,*k*), known as a carpal knob or spur. Carpal knobs are projecting bony cores used primarily in fighting, which in some taxa may have an outer layer of horn [50] but can be bare in others [51]. These rugose structures arise from the deposition of bone on the extensor process, to which they are fused. Carpal knobs and spurs occur in several anseriforms [51], most notably in steamer ducks [52] and anhimids [50]. Within anseriform species that bear them, they are better developed in males, but are still present in females [50]. Well-developed carpal knobs, similar to those of the male paradise shelduck *T. variegata*, were present in three specimens of our sample, which were also slightly larger compared to the rest (table 1). Both sexual dimorphism and age probably explain these differences [51]. Some of the specimens with the less protruding extensor process still displayed a form of rugose enlargement. From this we infer that, following the pattern in other anseriforms, those individuals of *W. tedfordi* with the well-developed carpal knobs are males. Anseriform species that bear prominent structures tend to engage in aggressive behaviour and hold year-round feeding and breeding territories [51]. The lack of well-developed carpal knobs in most of the specimens in our sample suggests that predominantly males may have engaged in aggressive behaviour. Howard [13] observed a ‘slight excrescence’ on the tip of the extensor process of *T. antiquus*, and illustrations clearly show [13], fig. 6, p. 16, a small carpal knob. This structure is not present in *P. pervetus* or the *Presbyornis* specimens from the Eocene of Mongolia [20], which closely resemble each other.

Compared with other presbyornithids, the morphology of the tarsometatarsus, with mediolaterally splayed trochleae for the articulation of the toes, a less plantarly retracted trochlea for the second digit, and a relatively low hallux, suggests that species of *Wilaru* were more terrestrial. Screamers, which have a similar tarsometatarsal morphology, are birds of predominantly terrestrial habits, frequenting open savannahs and wetlands (meadowlands, marshes, swamps and lakes with abundant vegetation) [53]. The tarsometatarsus is proportionally longer and more gracile in *P. pervetus*, in which the narrowly divergent trochleae and the markedly retracted trochlea for the second digit indicate more aquatic adaptations, as in most anseriforms (e.g. [17,44]). The length and most detail of the tarsometatarsus are not known for *T. antiquus*, but it appears to have resembled that of *P. pervetus* [17].

Terrestrial habits have evolved independently several times within Anseriformes [54,55], and even possibly within screamers [17,44] (§4.3). The terrestrial habits of species of *Wilaru* may, therefore, reflect a trophic specialization (such as herbivory) derived within Presbyornithidae. These differences are not surprising given the apparent temporal separation of *ca* 25 Ma between species of *Wilaru* and other presbyornithids, and may have been the key to the longevity of the presbyornithid lineage in Australia (§4.3).

The younger, larger and more robust *W. prideauxi* represents a further step from the morphology of *W. tedfordi* down the path of terrestriality. As such, species of *Wilaru* provide the first example for Australia of two successive species within an avian lineage in the Oligo-Miocene. Multiple lineages of mammals are known over this time period in the Namba and Etadunna Formations in Australia and form the foundation of the biochronological understanding of the different faunas [1,56]. The occurrence of *W. prideauxi* in the Ngama Local Fauna from Zone D of the Etadunna Formation at Lake Palankarinna, and in the Kutjamarpu Local Fauna from the Wipajiri Formation at Lake Ngapakaldi, South Australia, provides further evidence of the contemporaneity of these local faunas, otherwise linked by mammals, and supports their early Miocene age and distinction from underlying Etadunnan faunal zones [1].

### 4.3 The role of Gondwana and the evolutionary history of *Wilaru*

Several species of anatids were described from late Oligocene and early Miocene deposits of the Namba and Etadunna formations in South Australia [57], supporting an already established diversity of crown-group anatids by the late Oligocene. Having been recovered from the same late Oligocene and early Miocene localities, the survival of presbyornithids in Australia into the Neogene indicates they were living alongside crown-group anatids. Presbyornithids seem to have disappeared from the rest of the world during the Eocene [19], coinciding with the earliest records of stem group anatids. However, the more terrestrial adaptations of species of *Wilaru* suggest that in Australia, they may not have been in direct resource competition with coeval waterfowl. Similarly, at least two palaelodids and two species of flamingo cornered the wading niche in the lakes in which these deposits were formed [5,58]. The causes of the ultimate demise of species of *Wilaru* after the early Miocene are unknown, but as in the case of much of Australia’s fauna, climate change and the progressive aridification of the continent may have played a role, especially if species of *Wilaru* were territorial and dependent on specific habitats for breeding.

Fossils from the early Eocene Tingamarra Local Fauna, Queensland, were tentatively referred to the form-taxon Graculavidae (‘transitional shorebirds’ [59]) [32], but it has been acknowledged that some of the material may in fact be presbyornithid [30,32]. Indeed, the coracoid, fragment of humerus, and one distal tibiotarsus tentatively attributed to the ‘Graculavidae’ were recognized by Boles [32] as remarkably similar to *P. pervetus,* and we further note the marked similarity with the corresponding elements of *W. tedfordi* and other members of the Presbyornithidae. Further assessment of this material will help establish if presbyornithids have been in Australia since at least the early Eocene.

Australia’s long period of geographical isolation, from complete separation from Antarctica to a close proximity to the Indo-Malayan region (*ca* 50–15 [60,61]), has certainly promoted the extended temporal continuance of its fauna. The presence of presbyornithids in the early Miocene of Australia, therefore, ought not to appear all that surprising. Within mammals, marsupials and monotremes have survived in Australia since at least the early Eocene [62] and late Oligocene [63], respectively, long after most lineages disappeared elsewhere in the world (e.g. [64]). A similar pattern can be observed among birds, as the globally distributed Palaelodidae, which first appear in the fossil record during the early Oligocene, survived in Australia until the mid-Pleistocene [5], and the plains-wanderer lineage, which was once more widespread but has been on the continent since at least the late Oligocene [8,11], still remains in Australia with a sole representative, *Pedionomus torquatus*. Anseranatids, arguably known from the early Eocene and late Oligocene of Europe [19] and nowadays represented only by *A. semipalmata*, survive in Australia and New Guinea, having been recorded in Australia since the late Oligocene [7]. We note that although members of Anseranatidae are known from similar-aged deposits in Australia, they differ from presbyornithids in the morphology of most skeletal elements, but especially the humerus and coracoid.

Within Presbyornithidae, the postcranial morphology of *W. tedfordi* and *W. prideauxi*agrees with the South American *T. antiquus* in nearly all elements (§i), the tarsometatarsus being the exception. On the other hand, *P. pervetus* resembles *T. antiquus* in some features more than it does *W. tedfordi*, but mainly in the morphology of the tarsometatarsus. The overall close similarity between *W. tedfordi* and *T. antiquus* may lend support to the hypothesis that at least some aspects of the morphology of *P. pervetus* may be derived within Presbyornithidae (see also [19]). However, the uncertainty, at least for the time being, as to whether these similarities are derived or plesiomorphic both within Anseriformes and Presbyornithidae precludes a well-informed phylogenetic hypothesis. Assessing potential presbyornithid material from the Eocene of Australia, the discovery of cranial material of species of *Wilaru*, and the assessment of early-diverging anhimids (thought to be more *Presbyornis*-like [40]), may all contribute to clarifying the phylogenetic relationships between the different presbyornithid taxa.

Although conceiving a historical biogeographical scenario with the evidence at hand may be premature, the morphological similarity between species of *Wilaru* and *T. antiquus* emphasizes the role of Gondwana during the evolutionary history of the Presbyornithidae. This raises the possibility that members of the genera *Wilaru*, *Telmabates* and *Presbyornis* had a common ancestry on the southern landmasses, or at least that there was one Gondwanan radiation within Presbyornithidae including *T. antiquus* and species of *Wilaru*. Because only very few elements are known for the Cretaceous *T. gobiensis*, assessment of its position within Presbyornithidae, and the role Laurasia played during the early evolutionary history of presbyornithids will need to await the discovery of additional material. The morphology of *P. mongoliensis* and that of most elements attributed to *Presbyornis* sp. by Kurochkin & Dyke [20], some of which are probably attributable to *P. mongoliensis*, agrees well with the morphology of *P. pervetus*. A close relationship between the two can be explained by the geographical proximity of North America and Mongolia during the early Cenozoic [65].

### 4.4 Comments on presbyornithid relationships

There are marked differences between screamers and presbyornithids in major skeletal elements that extend beyond the highly pneumatic nature of anhimid limb bones, e.g. the humerus of anhimids has an inflated shaft and a pneumatic fossa pneumotricipitalis, the coracoid has a very reduced acrocoracoid with an unusually shallow sulcus supracoracoideus, and the carpometacarpus has a unique spur-like development of the processus extensorius. Despite this, many of the postcranial features we used in this study reflect interesting similarities with the Anhimidae, despite the derived aberrant morphology of this taxon [44]. Within anseriforms, characters that are shared between Anhimidae and Presbyornithidae, and not present in other Anseriformes (i.e. Anseranatidae and Anatidae), include (1) the presence of a deep fossa in the incisura capitis of the humerus; (2) both articular rims of the trochlea carpalis of the carpometacarpus extending caudally and distally to about same level; (3) a very elongate sulcus tendineus, extending to the distal end of the scar for m. extensor carpi ulnaris; (4) a very prominent linea intermuscularis cranialis of the femur, separated from the lateral margin of the crista trochanteris (in *P. pervetus* this line runs closer to crista trochanteris); (5) a much smaller, mediolaterally and proximodistally, medial condyle of the tibiotarsus compared to the lateral condyle; and (6) a hypotarsus with one well-developed sulcus hypotarsi and one well-marked crista intermedia hypotarsi with the rest reduced to a near flat embossment in the lateral portion of the hypotarsus (not present in *P. pervetus* and *T. antiquus*). Ericson [40] further noted the presence of pleurocoelous thoracic vertebrae in presbyornithids and screamers, but because of the pronounced pneumaticity in the skeleton of extant anhimids whether this feature is indeed homologous for the two remains to be ascertained.

Some of the characters listed above may be plesiomorphic for Anseriformes (e.g. characters 2, 3, and 5), but whether that is true of all features or whether some could be synapomorphic for a clade including (Anhimidae + Presbyornithidae) needs to be established in a cladistic framework. A yet undescribed Eocene representative of the Anhimidae may shed some light on the subject [66]. Although not formally described, this early Eocene specimen from Wyoming was briefly assessed by Ericson [40], who noted that several elements of the postcranial skeleton closely match those of presbyornithids, including the carpometacarpus, coracoid, furcula and tibiotarsus (see also [32]). *Chaunoides antiquus* from the late Oligocene–early Miocene of Brazil [67] is known from several fragmentary bones, and despite it being morphologically very similar to extant anhimids, the extreme pneumatisation of the skeleton that characterizes modern anhimids is absent. There is, therefore, a strong possibility that screamers are derived from presbyornithid-like birds or that they had a common ancestor. Although living screamers have a galliform-like hooked bill, it has been proposed that the rudimentary lamellae on the bill of anhimids indicates a secondary loss of the filter-feeding ability [44,68], and therefore the skull of early-diverging anhimids may have been more ‘anseriform-like’ than that of extant anhimids [40]. The occurrence of lamellae-like structures, however, may not necessarily be linked to filter-feeding, as aquatic herbivores may use these structures for grasping and cutting plants [54].

In any case, the recognition of *W. tedfordi* as a presbyornithid certainly calls for a reassessment of the phylogenetic position of Presbyornithidae within Anseriformes (see also [69–71]), which may be closer to the base of the anseriform tree than previously assumed [40,47,72]. Similarly, the phylogenetic placement of anseriform fossil taxa that have been based on cladistics analyses with limited taxon sampling and including *P. pervetus* alone, such as that of the Late Cretaceous *Vegavis iaai*from Antarctica [72], should be revised in the light of these new findings.

## 5. Conclusion

In this study we show that, contrary to previous reports, *W. tedfordi* from the late Oligocene of South Australia was not a burhinid (Charadriiformes), but a representative of the Presbyornithidae (Anseriformes). Additionally, we describe a slightly larger and more robust species of *Wilaru*, *W. prideauxi*, from the early Miocene of South Australia. This record extends the temporal continuance of presbyornithids by at least 25 million years, as they were believed to have disappeared from the fossil record by the early middle Eocene.

Unlike other presbyornithids, species of *Wilaru* were predominantly terrestrial birds, as indicated by the morphology of their tarsometatarsus. This adaptation likely contributed to their long-term survival in Australia, where they may have been present since at least the early Eocene, and where they lived alongside aquatic members of crown-group Anatidae. The presence of a bony excrescence on the extensor process of the carpometacarpus, linked with aggressive behaviour and also present in the presbyornithid *T. antiquus*and many extant anseriforms, may indicate that species of *Wilaru* were highly territorial.

The morphological similarity between species of *Wilaru* and the South American *T. antiquus* (§i) not only suggests a close relationship between the two, but also emphasizes the previously unexplored role of Gondwana in the evolutionary history of the Presbyornithidae, raising the possibility of a Gondwanan origin for the group, or at least a Gondwanan radiation. Similarly, the skeletal resemblance of the North American *P. pervetus* and the Mongolian *P. mongoliensis* (including specimens attributed to *Presbyornis* sp. from Mongolia) possibly indicates that these Northern Hemispheric species were more closely related to each other than to the Gondwanan *T. antiquus* and species of *Wilaru*. The phylogenetic affinities of *T. gobiensis* from the Late Cretaceous of Mongolia, here confirmed as a presbyornithid, remain obscure.

Although screamers (Anhimidae) may have evolved from presbyornithid-like birds, the uncertainty as to whether skeletal features shared by presbyornithids and anhimids are plesiomorphic within Anseriformes or indicative of a close relationship between the two cannot yet be resolved. In any case, recognition of *W. tedfordi* as a presbyornithid calls for a reassessment of the phylogenetic position of Presbyornithidae within Anseriformes.

## References

[1] Black KH, Archer M, Hand SJ, Godthelp H 2012 The rise of Australian marsupials: a synopsis of biostratigraphic, phylogenetic, palaeoecologic and palaeobiogeographic understanding. In *Earth and life*, pp. 983–1078. Dordrecht, The Netherlands: Springer.

[2] Flannery TF, Archer M, Richi TH, Jones R 1995 A new family of monotremes. *Nature* 377, 418–420. (doi:10.1038/377418a0)

[3] Scanlon SD, Lee MSY 2000 The Pleistocene serpent Wonambi and the early evolution of snakes. *Nature* 403, 416–420. (doi:10.1038/35000188) 10667791 10.1038/35000188

[4] Long JA, Archer M, Flannery TF, Hand S 2003 *Prehistoric mammals of Australia and New Guinea—100 million years of evolution*. Baltimore, MA: Johns Hopkins University Press.

[5] Baird RF, Vickers-Rich P 1998 *Palaelodus* (Aves: Palaelodidae) from the middle to late Cainozoic of Australia. *Alcheringa* 22, 135–151. (doi:10.1080/03115519808619196)

[6] Mourer-Chauviré C, Berthet D, Hugueney M 2004 The Late Oligocene birds of the Créchy Quarry (Allier, France), with a description of two new genera (Aves: Pelecaniformes: Phalacrocoracidae, and Anseriformes: Anseranatidae). *Senk. Leth.* 84, 303–315. (doi:10.1007/BF03043473)

[7] Worthy TH, Scanlon JD 2009 An Oligo-Miocene magpie goose (Aves: Anseranatidae) from Riversleigh, Northwestern Queensland, Australia. *J. Vert. Paleontol.* 29, 205–211. (doi:10.1671/039.029.0103)

[8] De Pietri VL, Scofield RP, Tennyson AJD, Hand SJ, Worthy TH In press Wading a lost southern connection: Miocene fossils from New Zealand reveal a new lineage of shorebirds (Charadriiformes) linking Gondwanan avifaunas. *J. Syst. Palaeontol.* (doi:10.1080/14772019.2015.1087064)

[9] Boles WE 1993 A logrunner *Orthonyx* (Passeriformes, Orthonychidae) from the Miocene of Riversleigh, north-western Queensland. *Emu* 93, 44–49. (doi:10.1071/MU9930044)

[10] Nguyen JM, Boles WE, Worthy TH, Hand SJ, Archer M 2014 New specimens of the logrunner *Orthonyx kaldowinyeri* (Passeriformes: Orthonychidae) from the Oligo-Miocene of Australia. *Alcheringa* 38, 245–255. (doi:10.1080/03115518.2014.861732)

[11] De Pietri VL, Camens AB, Worthy TH 2015 A plains-wanderer (Pedionomidae) that did not wander plains: a new species from the Oligocene of South Australia. *Ibis* 157, 68–74. (doi:10.1111/ibi.12215)

[12] Wetmore A 1926 Fossil birds from the Green River deposits of eastern Utah. *Ann. Carnegie Mus.* 16, 391–402.

[13] Howard H 1955 A new wading bird from the Eocene of Patagonia. *Am. Mus. Novit.* 1710, 1–25.

[14] Olson SL 1994 A giant *Presbyornis* (Aves: Anseriformes) and other birds from the Paleocene Aquia Formation of Maryland and Virginia. *Proc. Biol. Soc. Wash.* 107, 429.

[15] Tambussi CP, Noriega JI 1998 Registro de Presbiornítidos (Aves, Anseriformes) en sedimentitas de la Formación Vaca Mahuida (La Pampa, Argentina). *Asoc. Paleontol. Argent. pub. esp.* 5, 51–54.

[16] Benson RD 1999 *Presbyornis isoni* and other late Paleocene birds from North Dakota. *Smithson. Contrib. Paleobiol.* 89, 253–259.

[17] Ericson PG 2000 Systematic revision, skeletal anatomy, and paleoecology of the New World early Tertiary Presbyornithidae (Aves: Anaeriformes). *Paleo-Bios* 20, 1–23.

[18] Tambussi CP, Degrange FJ 2013 The Paleogene birds of South America. In *South American and Antarctic continental Cenozoic birds: paleobiogeographic affinities and disparities*. Springer Briefs in Earth System Sciences, pp. 29–47. Dordrecht, The Netherlands: Springer. (doi:10.1007/978-94-007-5467-6_5)

[19] Mayr G 2009 *Palaeogene fossil birds*. Heidelberg, Germany: Springer.

[20] Kurochkin EN, Dyke GJ 2010 A large collection of *Presbyornis* (Aves, Anseriformes, Presbyornithidae) from the late Paleocene and early Eocene of Mongolia. *Geol. J.* 45, 375–387.

[21] Kurochkin EN 1988 Cretaceous birds of Mongolia and their significance for the study [of the] phylogeny of class Aves. In *Fossil reptiles and birds of Mongolia* (ed. Kurochkin EN), pp. 33–42. Trudy Sovmestnoi Sovetsko-Mongol’skoi Paleontologicheskoi Ekspeditsii Moscow, Russia: Nauka. [In Russian.]

[22] Kurochkin EN 2000 Mesozoic birds of Mongolia and the former USSR. In *The age of dinosaurs in Russia and Mongolia* (eds MJ Benton, MA Shishkin, DM Unvin, EN Kurochkin), pp. 533–559. Cambridge, UK: Cambridge University Press.

[23] Eberle JJ, Greenwood DR 2012 Life at the top of the greenhouse Eocene world—a review of the Eocene flora and vertebrate fauna from Canada’s High Arctic. *GSA Bull.* 124, 3–23. (doi:10.1130/B30571.1)

[24] Harrison CJO, Walker CA 1976 Birds of the British upper Eocene. *Zool. J. Linn. Soc.* 59, 323–351. (doi:10.1111/j.1096-3642.1976.tb01017.x)

[25] Dyke GJ 2001 The fossil waterfowl (Aves: Anseriformes) from the Eocene of England. *Am. Mus. Novit.* 3354, 1–15. (doi:10.1206/0003-0082(2001)354<0001:TFWAAF>2.0.CO;2)

[26] Lydekker R 1891 *Catalogue of the fossil birds in the British Museum (Natural History)*. London, UK: British Museum (Natural History).

[27] Mayr G 2008 Phylogenetic affinities and morphology of the late Eocene anseriform bird Romainvillia stehlini Lebedinsky, 1927. *Neues Jahrb. Geol. Paläontol. Abh.* 248, 365–380. (doi:10.1127/0077-7749/2008/0248-0365)

[28] Kurochkin EN, Dyke GJ, Karhu AA 2002 A new presbyornithid bird (Aves, Anseriformes) from the Late Cretaceous of southern Mongolia. *Am. Mus. Novit.* 3386, 1–11. (doi:10.1206/0003-0082(2002)386<0001:ANPBAA>2.0.CO;2)

[29] Clarke JA, Norell MA 2004 New avialan remains and a review of the known avifauna from the Late Cretaceous Nemegt Formation of Mongolia. *Am. Mus. Novit.* 3447, 1–12. (doi:10.1206/0003-0082(2004)447<0001:NARAAR>2.0.CO;2)

[30] Elzanowski A, Boles WE 2012 Australia’s oldest Anseriform fossil: a quadrate from the Early Eocene Tingamarra Fauna. *Palaeontology* 55, 903–911. (doi:10.1111/j.1475-4983.2012.01166.x)

[31] Olson SL, Parris DC 1987 *The Cretaceous birds of New Jersey*. Washington, DC: Smithsonian Institution Press.

[32] Boles WE 1999 Early Eocene shorebirds (Aves: Charadriiformes) from the Tingamarra Local Fauna, Murgon, Queensland, Australia. *Rec. West. Aust. Mus. Supp.* 57, 229–238.

[33] Tedford RH, Archer M, Bartholomai A, Plane M, Pledge NS, Rich THV, Rich PV, Wells RT 1977 The discovery of Miocene vertebrates, Lake Frome area, South Australia. *BMR J. Aust. Geol. Geophys.* 2, 53–57.

[34] Rich PV, van Tets GF 1982 Fossil birds of Australia and New Guinea: their biogeographic, phylogenetic and biostratigraphic input. In *The fossil vertebrate record of Australasia* (eds PV Rich, EM Thompson), pp. 235–384. Clayton, Australia: Monash University Offset Printing Unit.

[35] Vickers-Rich P 1991 Avian fossils from the Quaternary of Australia. In *Vertebrate palaeontology of Australasia* (eds P Vickers-Rich, JM Monahan, RF Baird, TH Rich), pp. 721–808. Melbourne, Australia: Monash University Publications Committee.

[36] Boles WE, Finch MA, Hofheins RH, Vickers-Rich P, Walters M, Rich TH 2013 A fossil stone-curlew (Aves: Burhinidae) from the Late Oligocene/Early Miocene of South Australia. In *Paleornithological Research 2013: Proc. of the 8th Int. Meeting of the Society of Avian Paleontology and Evolution* (eds UB Göhlich, A Kroh), pp. 43–61. Vienna, Australia: Naturhistorisches Museum Wien.

[37] Baumel JJ, Witmer L 1993 Osteologia. In *Handbook of avian anatomy: nomina anatomica avium* (eds JJ Baumel, AS Kings, JE Breazile, HE Evans, JC Vanden Berge), pp. 45–132. Cambridge, MA: Nuttall Ornithological Club.

[38] Hardy JW 1959 A previously undescribed recurvirostrid from the Eocene of Utah. *Auk* 76, 106–108. (doi:10.2307/4081861)

[39] De Pietri VL, Scofield RP 2014 The earliest European record of a Stone-curlew (Charadriiformes, Burhinidae) from the late Oligocene of France. *J. Ornithol* 155, 421–426. (doi:10.1007/s10336-013-1022-8)

[40] Ericson PG 1997 Systematic relationships of the palaeogene family Presbyornithidae (Aves: Anseriformes). *Zool. J. Linn. Soc.* 121, 429–483. (doi:10.1111/j.1096-3642.1997.tb01286.x)

[41] Baker AJ, Pereira SL, Paton TA 2007 Phylogenetic relationships and divergence times of Charadriiformes genera: multigene evidence for the Cretaceous origin of at least 14 clades of shorebirds. *Biol. Lett.* 3, 205–209. (doi:10.1098/rsbl.2006.0606) 17284401 10.1098/rsbl.2006.0606 PMC2375939

[42] Mayr G 2011 The phylogeny of charadriiform birds (shorebirds and allies)—reassessing the conflict between morphology and molecules. *Zool. J. Linn. Soc.* 161, 916–934. (doi:10.1111/j.1096-3642.2010.00654.x)

[43] Feduccia A, McGrew PO 1974 A flamingo-like wader from the Eocene of Wyoming. *Rocky Mt. Geol.* 13, 49–61.

[44] Olson SL, Feduccia A 1980 *Presbyornis* and the origin of the Anseriformes (Aves: Charadriomorphae). *Smith. Contrib. Zool.* 323, 1–24.

[45] Rich TH *et al.* 1991 Australian Mesozoic and Tertiary terrestrial mammal localities. In *Vertebrate palaeontology of Australasia* (eds PV Rich, JM Monaghan, RF Baird, TH Rich), pp. 1005–1070. Melbourne, Australia: Pioneer Design Studio and Monash University Publications Committee.

[46] Megirian D, Prideaux GJ, Murray PF, Smit N 2010 An Australian land mammal age biochronological scheme. *Paleobiology* 36, 658–671. (doi:10.1666/09047.1)

[47] Livezey BC 1997 A phylogenetic analysis of basal Anseriformes, the fossil *Presbyornis*, and the interordinal relationships of waterfowl. *Zool. J. Linn. Soc.* 121, 361–428.

[48] Hackett SJ *et al.* 2008 A phylogenomic study of birds reveals their evolutionary history. *Science* 320, 1763–1768. (doi:10.1126/science.1157704) 18583609 10.1126/science.1157704

[49] Zelenkov NV, Kurochkin EN 2015 Class Aves. In *Fossil vertebrates of Russia and adjacent countries. Fossil reptiles and birds. Part 3* (eds EN Kurochkin, AV Lopatin, NV Zelenkov), pp. 86–290. Moscow, Russia: GEOS [In Russian.]

[50] Rand AL 1954 On the spurs on birds’ wings. *Wilson Bull.* 66, 127–134.

[51] Williams M 2015 Formidable carpal weaponry of *Anas chathamica*, Chatham Island’s extinct flightless duck. *Notornis* 62, 113–120.

[52] Livezey BC, Humphrey PS 1985 Territoriality and interspecific aggression in Steamer-Ducks. *Condor* 87, 154–157. (doi:10.2307/1367152)

[53] Carboneras C 1992 Screamers (Anhimidae). In *Handbook of the birds of the world alive* (eds J del Hoyo, A Elliott, J Sargatal, DA Christie, E de Juana). Barcelona, Spain: Lynx Edicions See http://www.hbw.com/node/52209 (on 7 October 2015).

[54] van der Leeuw AH, Kurk K, Snelderwaard PC, Bout RG, Berkhoudt H 2003 Conflicting demands on the trophic system of Anseriformes and their evolutionary implications. *Anim. Biol.* 53, 259–301. (doi:10.1163/157075603322539453)

[55] Olsen AM 2015 Exceptional avian herbivores: multiple transitions toward herbivory in the bird order Anseriformes and its correlation with body mass. *Ecol. Evol.* 5, 5016–5032. (doi:10.1002/ece3.1787) 26640679 10.1002/ece3.1787 PMC4662324

[56] Woodburne MO, MacFadden BJ, Case JA, Springer MS, Pledge NS, Power JD, Woddburne JM, Springer KB 1994 Land mammal biostratigraphy and magnetostratigraphy of the Etadunna formation (Late Oligocene) of South Australia. *J. Vert. Paleontol.* 13, 483–515. (doi:10.1080/02724634.1994.10011527)

[57] Worthy TH 2009 Descriptions and phylogenetic relationships of two new genera and four new species of Oligo-Miocene waterfowl (Aves: Anatidae) from Australia. *Zool. J. Linn. Soc.* 156, 411–454. (doi:10.1111/j.1096-3642.2008.00483.x)

[58] Miller AH 1963 The fossil flamingos of Australia. *Condor* 65, 289–299. (doi:10.2307/1365355)

[59] Feduccia A 1999 *The origin and evolution of birds*. New Haven, CT: Yale University Press.

[60] Hall BL 2009 Holocene glacial history of Antarctica and the sub-Antarctic islands. *Quat. Sci. Rev.* 28, 2213–2230. (doi:10.1016/j.quascirev.2009.06.011)

[61] Bijl PK *et al.* 2013 Eocene cooling linked to early flow across the Tasmanian Gateway. *Proc. Natl Acad. Sci. USA* 110, 9645–9650. (doi:10.1073/pnas.1220872110) 23720311 10.1073/pnas.1220872110 PMC3683727

[62] Beck RM, Godthelp H, Weisbecker V, Archer M, Hand SJ 2008 Australia’s oldest marsupial fossils and their biogeographical implications. *PLoS ONE* 3, e1858 (doi:10.1371/journal.pone.0001858) 18365013 10.1371/journal.pone.0001858 PMC2267999

[63] Woodburne MO, Tedford RH 1975 The first Tertiary monotreme from Australia. *Am. Mus. Novit.* 2588, 1–11.

[64] Sigé B, Archer M, Crochet JY, Godthelp H, Hand S, Beck R 2009 *Chulpasia* and *Thylacotinga*, late Paleocene-earliest Eocene trans-Antarctic Gondwanan bunodont marsupials: new data from Australia. *Geobios* 42, 813–823. (doi:10.1016/j.geobios.2009.08.001)

[65] Brikiatis L 2014 The De Geer, Thulean and Beringia routes: key concepts for understanding early Cenozoic biogeography. *J. Biogeogr.* 41, 1036–1054. (doi:10.1111/jbi.12310)

[66] Houde P 1996 A fossil screamer from the Eocene of Wyoming (Anseriformes: Anhimidae). In *Program and Abstracts, 4th Int. Meeting of the Society of Avian Paleontology and Evolution*, *Washington, DC, 4–7 June 1996*.

[67] Alvarenga HMF 1999 A fossil screamer (Anseriformes: Anhimidae) from the middle Tertiary of Southeastern Brazil. *Smith. Contrib. Paleobiol.* 89, 223–230.

[68] Parker WK 1863 On the systematic position of the crested screamers (Palamedea Chavaria). *Proc. Zool. Soc. Lond.* 4, 511–518.

[69] Elzanowski A, Stidham TA 2010 Morphology of the quadrate in the Eocene anseriform Presbyornis and extant galloanserine birds. *J. Morphol.* 271, 305–323. 19806655 10.1002/jmor.10799

[70] Zelenkov NV 2011 Morphological hemiplasies in cladistic studies of phylogeny (with examples from birds). *Biol. Bull.* 38, 905–911. (doi:10.1134/S106235901109010X)

[71] Elzanowski A 2014 More evidence for plesiomorphy of the quadrate in the Eocene anseriform avian genus *Presbyornis*. *Acta Palaeontol. Pol.* 59, 821–825.

[72] Clarke JA, Tambussi CP, Noriega JI, Erickson GM, Ketcham RA 2005 Definitive fossil evidence for the extant avian radiation in the Cretaceous. *Nature* 433, 305–308. (doi:10.1038/nature03150) 15662422 10.1038/nature03150

