# Correction to ‘The unexpected survival of an ancient lineage of anseriform birds into the Neogene of Australia: the youngest record of Presbyornithidae’

**DOI:** 10.1098/rsos.201430

**Published:** 2020-11-11

**Authors:** Vanesa L. De Pietri, R. Paul Scofield, Nikita Zelenkov, Walter E. Boles, Trevor H. Worthy

*R. Soc. Open Sci.* **3**, 150635. (Published online 1 February 2016) (doi:10.1098/rsos.150635)

This publication was not registered with Zoobank. Because the work must be registered in Zoobank before it is published [1], the name *Wilaru prideauxi* found in De Pietri *et al*. [2] was unavailable. This correction has been issued to rectify this omission and make the name available in terms of nomenclature and provide the following Registration Zoobank ID: urn:lsid:zoobank.org:pub:1BF287FE-4D10-4E22-A0C5-9CC0126EA259. Description and comparisons for this taxon can be found in De Pietri *et al*. [2].

*Wilaru prideauxi* sp. nov.

Zoobank ID: urn:lsid:zoobank.org:act:FE6BAC76-C919-4F56-A847-7CA3CA96F290

*Holotype:* Right tarsometatarsus SAM P.53136 (formerly UCMP 108052) (figures in De Pietri *et al*. [2]).

*Type locality and age:* Leaf Locality, Lake Ngapakaldi, South Australia (UCMP locality V6213); Wipajiri Formation; Kutjamarpu Local Fauna; *ca* 23.4–22 Ma.

*Etymology:* After vertebrate palaeontologist Gavin Prideaux (1969–), who has worked extensively on Oligo-Miocene mammalian faunas from South Australia, including the formations bearing fossils of species of *Wilaru*.

*Differential diagnosis:* Only slightly larger than *Wilaru tedfordi* but considerably stouter (figs 1 and 2 in De Pietri *et al*. [2]). Differs from *W. tedfordi* in: tarsometatarsus with (i) sulcus extensorius shallower; (ii) plantarly, rounded ligamental scar between trochleae metatarsorum [note this term is here corrected from ‘metatarsi’ in De Pietri *et al*. [2] II and IV deeper and closer to foramen vasculare distale; (iii) fossa metatarsi nearly absent. Carpometacarpus with (iv) synostosis metacarpalis distalis proximodistally shorter; (v) facies articularis digitalis minor projecting further distally.

*Remarks:* For description, comparisons and figures, see De Pietri *et al*. [2].

Note also that the caption of figure 1 in De Pietri *et al*. [2] erroneously states that figure 1*d*, a distal left humerus (paratype AMNH 11452) of *W. tedfordi* Boles, Finch, Hofheins, Vickers-Rich, Walters, & Rich, 2013, is shown in caudal view. It is shown in cranial view.
